# 2015: Year of Transition

**DOI:** 10.5935/1678-9741.20150090

**Published:** 2015

**Affiliations:** 1 Editor-in-Chief BJCVS E-mail: domingo@braile.com.br

The Brazilian Journal of Cardiovascular Surgery (BJCVS) initiated a series of changes in 2015 aimed at celebrating its 30^th^ anniversary in 2016. The most important ones were the adoption of English as the official language and the change of periodicity from quarterly to bimonthly, with two more regular annual editions. Every transition brings expectations, but despite the difficulties that crop up during this process, I believe we can follow the path without too many mishaps, thanks to the efforts of the Editorial Board, Associate Editors, Editorial Council and staff and Board of Directors of the Brazilian Society of Cardiovascular Surgery (BSCVS) which gave essential support for us to begin and continue this process.

We have removed the rest of the content in Portuguese still present in the magazine (title, abstract and key words and name in Portuguese - Revista Brasileira de Cirurgia Cardiovascular) since the volume 30.5, making it definitely a periodical only in English. I repeat that English is the official language of science in the world and if we want to become part of the mainstream of scientific communication, the required standards must be followed.

Our website (www.bjcvs.org) is also undergoing revision and updating in order to be more interactive and "friendly", facilitating the process of submission and review of the manuscripts. We intend to make it more attractive and, therefore, glean new readers and authors interested in submitting their manuscripts.

Let's celebrate our 30 years of uninterrupted publications and services provided to the Brazilian cardiovascular surgery and related fields the way this date deserves it. The 43^rd^ Congress of the BSCVS, to be held April 7-9 in Fortaleza, CE, Brazil, will have part of its programming dedicated to the BJCVS, and certainly will please the participants with whom we will gladly share our 30^th^ year anniversary celebration.

In all 2016 issues, we are going to publish comments of Directors of the BSCVS, Departments and its Members, as well as all of our Editorial Board and BSCVS partners who wish to contribute their exegesis regarding this significant date.

## THE REASON WHY WE CELEBRATE 30 YEARS OF EXISTANCE

"A bit more than one hundred years ago, early last century, the life expectancy of Brazilians was merely 30 years. The average was pulled down by infant mortality and diseases for which there was no treatment at that time, such as typhoid fever, yellow fever, tuberculosis, leprosy, etc.

The corporate world has a remarkable resemblance to this picture. A survey carried out by the Instituto Brasileiro de Planejamento Tributário (Brazilian Institute of Tax Planning - BIPT) exclusively for Revista Época Negócios reveals that companies often become extinct when they are about 30 years old!" The BJCVS is going through this critical phase renewing itself every issue.

Freely adapted from the article: "Por que as empresas vivem tão pouco?" "Why do companies live so little?" (Title in English)^[1]^ [Marcelo Cabral, Revista Época Negócios] January 2014.

http://goo.gl/jlMdeI

## ECMO

Among the articles in this issue, approaching various aspects of our specialty, I highlight "ECMO: Improving our Results by Chasing the Rabbits" (page 657), by Dr. Luiz Fernando Canêo and Dr. Rodolfo Neirotti. The authors brilliantly describe the benefits of using this device, created 37 years ago, but little use in Latin American countries, including Brazil. They also show the work of the Extracorporeal Life Support Organization (ELSO) to compile global data on the use of ECMO and support the training of multidisciplinary teams in using this equipment, valuable for saving lives.

Also showing the difficulties in the use of ECMO in Brazil, we published the letter of Dr. Helmgton Souza, titled "Circulatory Assistance: the Challenges of Technological Incorporation in Brazil" (page 676). He highlights the delay to implement the new medical technologies in our country due to barriers imposed, citing the example of ECMO, still considered "experimental", bringing a number of limitations to professionals who intend to use it. Dr. Souza encourages those involved in the issue to engage in dialogue, so that the technologies can be incorporated into the daily routines.

## CME

In this BJCVS issue, the following articles are available to test the Continuing Medical Education (CME): "*Effects of Cardiopulmonary Bypass on Mediastinal Drainage and the Use of Blood Products in the Intensive Care Unit in 60- to 80-Year-Old Patients Who Have Undergone Coronary Artery Bypass Grafting" (page 597); "Application of Mechanical Ventilation Weaning Predictors After Elective Cardiac Surgery" (page 605); "Alternative Physical Therapy Protocol Using a Cycle Ergometer During Hospital Rehabilitation of Coronary Artery Bypass Grafting: a Clinical Trial" (page 615) and "Efficacy Analysis of a Script-based Guide for EVAR Execution: is It Possible to Reduce Patient Exposure to Contrast, Operative Time and Blood Loss even when Advanced Technologies are not Available?" (page 650).*

It's important to remember that 29.4 - 30.3 issue tests will be worth up to 5 points for the Specialist Title in 2016. The vouchers should be sent via email to the BSCVS until February 12, 2016. More information can be obtained by accessing the link:

http://www.bjcvs.org/cme/

This can be summarized to:

http://goo.gl/rWFapN

using google application:

https://goo.gl/

### Social networks

Besides the print and online editions, the complete contents of the BJCVS can be accessed on devices (smartphones, tablets, etc.) using Android and iOS systems. In addition to that, in order to seek greater visibility, we are also present on Facebook and Twitter for short notes, respectively:

https://goo.gl/nmox5q

https://goo.gl/MCwXgF

Following the example of high impact journals, we are also providing a blog, which has been proved very useful.

http://goo.gl/YLJvuI

These social media sites always bring news of our magazine, as well as cardiac surgery themes and scientific publications in general.

I draw readers' attention to the importance of collaboration of all, so that these media are accessed and, furthermore, that can be a meeting point to disseminate ideas and suggestions.

## BSCVS

I will take advantage of this great opportunity to congratulate the Board of the 2014 - 2015 BSCVS, chaired by Dr. Marcelo Matos Cascudo, who spared no effort to enhance our specialty and materialize the old dream of having its own headquarter, wider, with the possibility to implement refresher courses for cardiovascular surgeons, keeping us updated on the state-of-theart surgical procedures.

I wish good luck to those who will conduct our Society in the biennium 2016-2017, led by Dr. Fabio Jatene^[2]^, currently Full Professor of Cardiovascular Surgery at Faculdade de Medicina da Universidade de São Paulo, and Director of the Cardiovascular Surgery Division at Instituto do Coração, in São Paulo, SP, Brazil, a very competent colleague with merits already proven in multiple activities. He has been part of the BSCVS for many years, having occupied almost all Board positions, besides being the Editor-in-Chief of the BJCVS, preceded by the unforgettable Prof. Adib Jatene^[3]^.

I thank BSCVS, advertisers and partners for believing in BJCVS, enabling our work to be developed this year, despite the immense difficulties that our country is experiencing with consequences for all sectors of society.

I insist on being optimistic and believe that the strength of the good citizens, who live in Brazil, will be greater than the crisis and this will hopefully get us back on track. We recall the lesson of our patron, the late Professor Zerbini, whom we all miss so dearly: "Nothing resists the work^[4]^." So we will win!

May Christmas renew our hopes and a Happy 2016 to all of us!

Editor-in-Chief BJCVS E-mail: domingo@braile.com.br
